# Development of a Continuous System for 2-Phenylethanol Bioproduction by Yeast on Whey Permeate-Based Medium

**DOI:** 10.3390/molecules26237388

**Published:** 2021-12-06

**Authors:** Karolina Drężek, Joanna Kozłowska, Anna Detman, Jolanta Mierzejewska

**Affiliations:** 1 Chair of Drug and Cosmetics Biotechnology, Faculty of Chemistry, Warsaw University of Technology, 00-664 Warsaw, Poland; karolina.drezek@pw.edu.pl (K.D.); kozlowska.joanna.1997@gmail.com (J.K.); 2 Laboratory of White Biotechnology, Institute of Biochemistry and Biophysics, Polish Academy of Sciences, 02-106 Warsaw, Poland; annadetman@ibb.waw.pl

**Keywords:** 2-phenylethanol, whey permeate, yeast, *Kluyveromyces*, continuous culture, batch culture, bioreactor

## Abstract

2-Phenylethanol (2-PE) is an alcohol with a rosy scent and antimicrobial activity, and therefore, it is widely used in the food and cosmetic industries as an aroma and preservative. This work was aimed to draw up a technology for 2-PE bioproduction on whey permeate, which is waste produced by the dairy industry, rich in lactase and proteins. Its composition makes it a harmful waste to dispose of; however, with a properly selected microorganism, it could be converted to a value-added product. Herein, two yeast *Kluyveromyces marxianus* strains and one *Kluyveromyces lactis*, isolated from dairy products, were tested for 2-PE production, firstly on standard media and then on whey permeate based media in batch cultures. Thereafter, the 2-PE bioproduction in a continuous system in a 4.8 L bioreactor was developed, and subsequently, the final product was recovered from culture broth. The results showed that the yield of 2-PE production increased by 60% in the continuous culture compared to batch culture. Together with a notable reduction of chemical oxygen demand for whey permeate, the present study reports a complete, effective, and environmentally friendly strategy for 2-PE bioproduction with a space-time yield of 57.5 mg L^−1^ h^−1^.

## 1. Introduction

In recent years, rapid development of technologies that use organic waste to produce various molecules, such as biofuels, aromas, and enzymes, has been observed [1,2,3]. Three main types of organic wastes worldwide are being processed, i.e., the lignocellulosic biomass, vegetable or fruit pulp, and whey/whey permeate, which are major byproducts of the dairy industry. Due to the high concentration of lactose and the presence of other organic substances in it, whey has a high organic load causing, consequently, a high COD (chemical oxygen demand) value (60–80 g L^−1^) and making it an environmental problem. Whey’s annual production is estimated to be over 160 million tons, with a predictable growth rate of 1–2% yearly [4]. Processing of the dairy wastes to various biomolecules has proved to be a profitable option, both by reducing the costs of the manufacturing or other treatment processes required for its disposal. The most often, whey is subjected to ultrafiltration to recover proteins from it, while the whey permeate that still contains large amounts of lactose and minerals is spray dried and can be used by the food industry. However, the amount of whey permeate is still significant for management and work is currently underway to use it in biorefineries [5]. Yet, in order to utilize the lactose and the inorganic compounds present in whey permeate, it is crucial to select an appropriate strain of the microorganism. The ideal candidate seems to be yeast of the *Kluyveromyces* genus, especially *K. marxianus*, which has an appropriate trait for industrial applications, namely a high temperature- and acid-tolerance, as well as a short doubling time [6,7,8]. Moreover, unlike classic brewer’s yeast *Saccharomyces cerevisiae*, some *Kluyveromyces* species, e.g., *K. marxianus* and *K. lactis*, can utilize lactose, thanks to the presence of the genes encoding a lactose permease and a β-galactosidase [6]. Noteworthy, the long history of safe application in foods enabled *K. marxianus* and *K. lactis* to achieve GRAS (Generally Recognized As Safe) and QPS (Qualified Presumption of Safety) status in the United States and European Union, respectively.

Recent studies have shown that *K. marxianus* produces various compounds such as esters and fusel alcohols with a pleasant fruit or honey flavor applicable in the food industry [8]. Among others, yeast strains producing significant amounts of 2-phenylethanol (2-PE) have been selected [9]. 2-PE is an aliphatic alcohol which, in addition to the rose fragrance, also has antibacterial and antifungal properties [10,11,12]. This makes it a valuable additive that not only gives an attractive fragrance, but also protects the product against rapid deterioration caused by the growth of undesired microorganisms. Although the biotechnological production of 2-PE has been studied for over 20 years, there is still no route to its chemical synthesis that is economically competitive. Therefore, to reduce the costs of 2-PE bioproduction, various wastes from the food and agri-cultural industry have been tested as a growth medium for microorganisms [13,14].

To date, there are a few publications indicating the possibility to utilize whey, but not whey permeate, as a culture medium for the microbial production of 2-PE [15,16,17]. Thus, the research direction on the bioproduction of 2-PE on whey permeate is very interesting, but the key is to answer three basic questions: (i) will it be possible to develop an efficient bioprocess to produce 2-PE on whey permeate (ii) and then to isolate and purify the desired aroma using standard techniques, e.g., extraction and distillation? and (iii) will the proposed idea of diary waste management reduce its COD ratio and, thus, make it less harmful to the environment? An attempt to answer these questions is presented within this paper. The main goal was a detailed study of utilization of whey permeate for the microbiological production of 2-PE. Herein, all stages of the bioproduction of a chosen aroma were investigated, from selecting efficient yeast producers, through optimization of the physico-chemical bioprocess parameters, to product purification. 

## 2. Results and Discussion

### 2.1. Evaluation of the Potential of Newly Isolated Yeasts for 2-PE Production in Well-Defined Media

Yeast can produce 2-PE through de novo synthesis or bioconversion of L-phenylalanine (L-phe). The first route does not require the addition of a precursor to the culture medium. In this work, the three *Kluyveromyces* strains, isolated from dairy products, were also tested for the de novo synthesis of 2-PE in a standard Sabouraud medium (SAB) dedicated to cultivation of microscopic fungi. During a 48 h incubation at 30 °C, all yeast strains synthesized 2-PE, but a higher productivity was obtained for *K. marxianus* WUT216 and WUT240 (0.26 ± 0.02 and 0.30 ± 0.03 g of 2-PE L^−1^) than for *K. lactis* WUT175 (0.13 ± 0.01 g of 2-PE L^−1^). However, in many yeasts, including the present strains, produce 2-PE de novo, the final concentration of 2-PE in the culture broth remains low and cannot be the route to economically viable bioprocesses [18].

The production of 2-PE in yeast can be greatly increased by adding L-phe to the medium. Hence, in the next step, the biotransformation efficiency of newly isolated *Kluyveromyces* strains was evaluated in medium 8 (previously applied for testing the 2-PE production by series of yeasts [16]) at various temperatures. The highest concentrations of 2-PE were achieved in the cultures of all three tested yeasts conducted at 25 °C, and generally, the productivity dropped down at higher temperatures (Table 1). Only for the cultures of *K. marxianus* WUT240, there was not a significant difference (less than 10%) in the obtained 2-PE titers at 25 °C and 30 °C. Consistent with de novo synthesis, *K. lactis* WUT175 also produced less 2-PE through L-phe biotransformation in medium 8 than *K. marxianus* strains.

**Figure 1:**
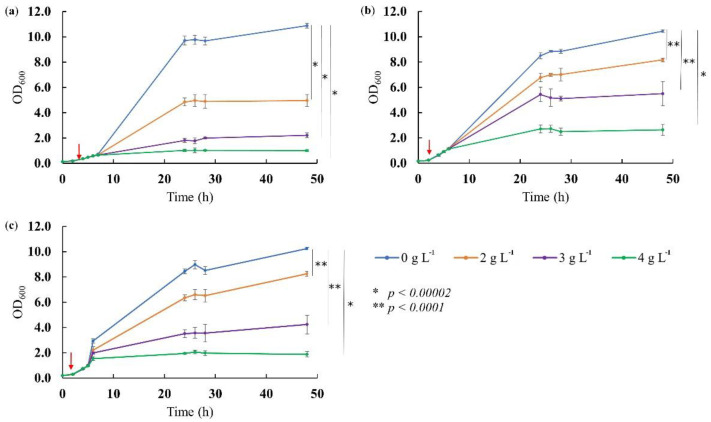
Effect of the presence of exogenous 2-PE on the growth of (**a**) *K. lactis* WUT175, (**b**) *K. marxianus* WUT216, and (**c**) *K. marxianus* WUT240. For each strain, four 48 h batch cultures in SAB were conducted. When the cultures reached OD_600_ values of 0.6–0.8 (indicated by a red arrow), 2-PE was added to three cultures to final concentrations of 2, 3, and 4 g L^−1^, respectively. The fourth culture was not supplemented with 2-PE and served as a control. Analyses were performed in triplicate and data are presented as the mean ± SD. Statistical significance between values is marked with asterisks.

Biotechnological production of 2-PE is limited by its inhibiting effect on the growth of the microorganism. Various yeasts show different resistance to 2-PE; however, it has been repeatedly reported that a concentration above 1.4 g L^−1^ is toxic and affects 2-PE synthesis [3,19,20]. Therefore, it is a challenging goal to find a yeast that can produce significant amounts of 2-PE while being resistant to its high concentrations. To check the limiting range of 2-PE titer for the WUT175, WUT216, and WUT240 strains, the cultures were conducted in the presence of the exogenous 2-PE. The study revealed that *K. lactis* is much less resistant to 2-PE than *K. marxianus* (Figure 1).

The concentration of 3 g L^−1^ 2-PE decreased the final OD_600_ of the WUT175 culture by 80%, while the WUT216 strain by 59% and the WUT240 strain by 48%. When yeasts were cultured in the medium containing 2 g L^−1^ of 2-PE, the growth of WUT175 by more than 55%, while WUT216 and WUT240 strains were inhibited by only ~20%. A similar study carried out on *K. marxianus* CCT 7735 revealed that the concentration of 2.5 g L^−1^ limited the growth of the strain by 64% [21]. Thus, the WUT216 and WUT240 strains are less fragile to 2-PE than the previously reported one. Taking into account that in case of *K. marxianus* CCT 7735, a titter of 3.44 g L^−1^ 2-PE in batch cultures at 30 °C after optimization procedure was obtained [21], it is likely that under optimized conditions, it will also be possible to increase the production of 2-PE in the WUT216 and WUT240 strains.

### 2.2. 2-PE Production on Whey Permeate Based Media in Batch Cultures: In Shaking Flasks and a 4.8 L Bioreactor

Cheese dairies generate enormous amounts of whey, from which they recover mainly proteins through ultrafiltration. The resulted whey permeate is then spray dried and sold e.g., as a feed or food additive. However, dairies do not have an outlet for all this whey permeate. Hence, it is worth looking for other applications for this waste.

In this work, commercially available dried whey permeate containing, among others, approx. 80% of lactose, was used as a source of carbon and micro-/macro-nutrients to prepare two media, WP1 and WP2, for *K. marxianus* and *K. lactis* strains to produce 2-PE. Specifically, 1.5% and 2.5% aqueous solutions of whey permeate, containing, respectively, approx. 12 g and 20 g L^−1^ of lactose, and supplemented with L-phe were tested in batch cultures in shaking flasks. Higher concentrations of whey permeate were not used to avoid the fermentation of lactose to ethanol by yeasts, since it is known from previous reports that the presence of ethanol enhances the toxic effect of 2-PE for *S. cerevisiae* [22]. Consequently, the yield of 2-PE production could be adversely affected by the increasing concentration of ethanol in the cultures of *Kluyveromyces* strains. The conducted research revealed that both WP1 and WP2 media were suitable for 2-PE production, and the highest titer of around 2 g L^−1^ was achieved for each culture (Table 2). In the case of the *K. lactis* WUT175 strain, even a significantly increased production of 2-PE was observed in whey permeate-based media compared to the cultures in medium 8 (e.g., at 25 °C 2.12 ± 0.19 vs. 1.63 ± 0.01 g L^−1^).

The next research task was to scale up the production of 2-PE on whey permeate from batch flask cultures to a 4.8 L bioreactor. Based on the initial screening in the shaking flasks, *K. marxianus* WUT240 strain was chosen as the best producer, having both the highest resistance to changing conditions (2-PE production in the range of 25–30 °C was almost identical), showing good tolerance to exogenous 2-PE and biosynthesizing 2-PE in WP2 medium with the highest yield. The first three batches in the bioreactor were conducted at 25 °C, with changing air flow rate and stirring speed to ensure adequate oxygenation. None of the tested parameters significantly affected 2-PE production, the 2-PE titer varied in a range of 1.66 and 1.80 g L^−1^ (Table 3). However, along with the improvement of the oxygenation in the bioreactor, there was a noticeable decrease in the amount of the ethanol produced. Nevertheless, under tested conditions, the loss in the 2-PE production yield of 16% with respect to shaking flask cultivation was observed; therefore, the temperature change from 25 to 30 °C in further experiments was applied. After increasing the temperature, a significant enhancement in the production of 2-PE, 2.59 g L^−1^ vs. 1.88 g L^−1^ (rise of 44%), was obtained. Simultaneously, there was a substantial improvement in L-phe utilization (increase of 32%), the key substrate in culture medium.

### 2.3. Continuous System for 2-PE Production on Whey Permeate Based Medium

Batch culture, while allowing for easy control of process conditions, has its efficiency limitations. In the case of 2-PE production, it is mainly the toxic nature of 2-PE on the yeast, which cannot be eliminated with this type of culture. To increase production yield, it is necessary to apply other bioprocess mode or in situ product recovery technique (ISPR). There are already some reports on the ISPR methods being used [23,24,25,26,27], yet fed-batch or continuous cultures were not often considered [14,28,29].

**Figure 2:**
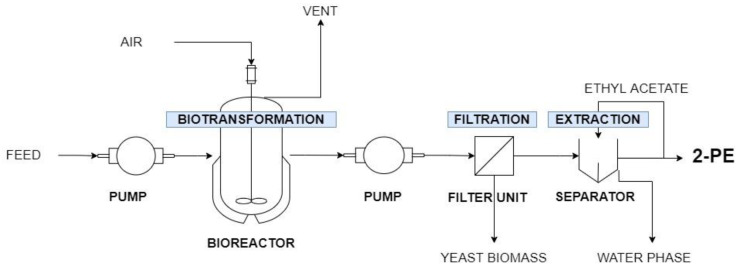
Schematic representation of continuous system for 2-PE bioproduction and its recovery from fermentation broth.

In this work, continuous approach to enhance the bioproduction of 2-PE in whey permeate residue was evaluated (Figure 2). Based on the best variant from batch cultures conducted in the bioreactor, temperature, air flow rate, and stirring speed were set at 30 °C, 1.5 L min^−1^, and 250 rpm, respectively. Steady states were obtained during chemostats at dilution rates of 0.05 or 0.1 h^−1^, namely continuous 1 and continuous 2 systems. This was selected based on literature data on continuous breeding of *K. marxianus* in other bioprocess [30]. The 2-PE produced by yeasts was recovered from the fermentation broth by ethyl acetate extraction.

Herein, the use of continuous culture was successful and allowed to significantly increase key process indicators (Table 4). Moreover, the dilution rate has been found to have a great impact on the production yield. During cultivation, the 2-PE concentration in the bioreactor remained at moderate concentration (around 1 g L^−1^) without adversely affecting the WUT240 strain in both tested variants. At a higher dilution rate (0.1 h^−1^), a lower amount of 2-PE was obtained. Namely, from 72 h culture, 5.63 ± 0.54 g of 2-PE was extracted, whereas at the same time, 8.29 ± 0.82 g of 2-PE was obtained when the dilution rate was set at 0.05 h^−1^ (32% difference). Compared to the results determined for the batch cultures, an increase of 8% and 60% in 2-PE production yield (Y_2-PE_) was gained. In both tested variants, lactose was completely utilized, and ethanol was not detected in medium by HPLC analysis. Simultaneously, there was a considerable increase in L-phe utilization; 27% (0.1 h^−1^) and 31% (0.05 h^−1^) with respect to batch mode.

**Figure 3:**
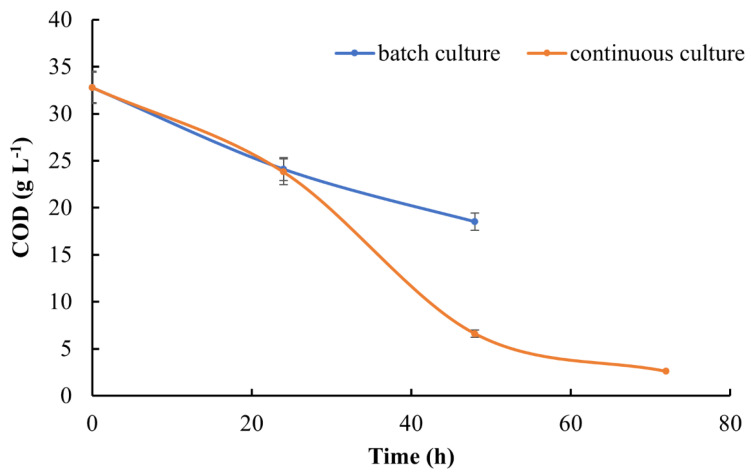
Reduction of COD load in *K. marxianus* WUT240 cultures over 48 h batch and 72 h continuous operation in a 4.8 L bioreactor.

Furthermore, during cultivation in bioreactors both under batch and continuous mode, every 24 h samples of culture broth were collected, and the COD parameter was determined. The results obtained proved that the proposed idea of whey permeate management makes it less harmful to the environment and significantly reduces its COD load. However, better results were obtained when continuous mode was applied. An initial COD of 33 g L^−1^ was minimized to 18.5 g L^−1^ in 48 h batch cultures and 2.6 g L^−1^ in the continuous system (Figure 3).

Thus, using whey permeate as a culture medium for *Kluyveromyces* strains, it is not only possible to develop an efficient process, but also to propose a promising way for its management and reduction of harmful COD loads.

## 3. Materials and Methods

### 3.1. Isolation, Identification, and Physiological Characterization of Yeast Strains

Samples of kefyr and mare’s milk were spread on standard Sabouraud agar plates (SAB, Merck, Darmstadt, Germany) with antibiotics (ampicillin and streptomycin at a final concentration of 100 and 20 mg L^−1^, respectively). The plates were incubated for 2–3 days at 30 °C. The single colonies grown on plates were then streaked on fresh SAB, and once again incubated 2–3 days at 30 °C. Then, from the obtained pure cultures of three new strains, total genomic DNA was isolated [31] and specific fragments (the ITS1-5.8S-ITS2 regions and the D1/D2 domains of 26S rDNA) were amplified by PCR with a pair of ITS1F (5’TCCGTAGGTGAACCTGCGG3’) and ITS4R (5’TCCTCCGCTTATTGATATGC3’) or NL4R (5’GGTCCGTGTTTCAAGACGG3’) and NL1F (5’GCATATCAATAAGCGGAGGAAAAG3’) primers, respectively [32]. Products of reactions were sequenced by Genomed S.A. (Genomed SA, Warsaw, Poland). Obtained DNA sequences were deposited in the NCBI GenBank database (WUT175: OK093387, OK093398; WUT216: OK093388, OK093399; WUT240: OK093389, OK093400) and used in phylogenetic analysis, according to [33]. Based on the DNA homology to known yeast species, three newly isolated strains were assigned to *K. lactis* WUT175, *K. marxianus* WUT216, and *K. marxianus* WUT240.

The isolates were also investigated by conventional identification methods based on the observation of the morphology, the utilization of carbon sources, the range of growth temperature and the extracellular enzymatic activity [34,35]. Yeasts were deposited in a publicly available Warsaw University of Technology Yeast Collection (WUT YC) and all data can be found on www.wutyeastcollection.pw.edu.pl (accessed on 3 November 2021) and Table S1.

### 3.2. Batch Cultures for 2-PE Production in Shaking Flasks

A sterile loop full of biomass from single colonies was used to inoculate 5 mL of SAB medium (Merck) in 15 mL glass test tubes and grown overnight at 30 °C. For the experiments in 100 mL flasks, 1 mL of overnight culture was diluted in 25 mL of selected medium: 8, SAB or whey permeate-based WP1 and WP2 (Table 5). Batch cultures were incubated at 25, 30 or 37 °C with shaking at 240 rpm (LAB Companion SI-600R, Ramsey, MN, USA) for 48 h in triplicate.

### 3.3. Yeast Growth in the Presence of Exogenous 2-PE

The effect of exogenous 2-PE supplementation on yeast cells was tested in accordance with [13]; instead of YPD, the SAB medium was used.

### 3.4. Batch Cultures for 2-PE Production in WP2 Medium in a 4.8 L Bioreactor

An overnight culture of the *K. marxianus* WUT240 strain in SAB medium was used to inoculate 2 L of WP2 medium in a 4.8 L bioreactor (Applikon ez-control, Applikon Biotechnology, Schiedam, The Netherlands) equipped with pH, temperature, and dissolved oxygen control. The batch cultures were conducted for 48 h at 25 or 30 °C with a stirring speed of 250 or 500 rpm and air flow rate maintained at 0.75 or 1.5 vvm (gas volume flow per unit of liquid volume per minute) (details included in Table 6).

Then, 1 mL samples were collected in duplicate at indicated time points to determine OD_600_, lactose, L-phe, and 2-PE concentration. Additionally, after 24 h, 48 h 10 mL samples were collected to determine the dry cell weigh (DCW) and chemical oxygen demand (COD).

### 3.5. Continuous Experimental System for 2-PE Biotransformation

A 4.8 L bioreactor equipped with pH, temperature, and dissolved oxygen control was used for all reactor runs. The aeration rate was controlled at 0.75 vvm (air flow rate of 1.5 L min^−1^) and the stirring speed set at 250 rpm. The temperature was controlled at 30 °C. The initial pH was 5 and it was not stabilized during cultivation. To inoculate 2 L of WP2 medium and start the bioprocess, 2% inoculum, an overnight culture of the WUT240 strain in SAB medium, was used. The yeasts were grown for 24 h before the medium flow was started. At that point, 0.5% (*w/v*) whey permeate solution was dosed into the bioreactor at a flow of 1.7 mL min^−1^ or 3.3 mL min^−1^ (Lead Fluid BT50S peristaltic pump) and the culture broth was pumped out the fermenter. In this way, the dilution rate was set at 0.05 h^−1^ or 0.1 h^−1^ in continuous 1 and continuous 2 system, respectively. The culture fluid was applied to a funnel with a cellulose filter in order to separate the biomass and in 24 h cycles subjected to 2-PE extraction (Figure 2). Samples were taken out of the reactor for OD_600_, DCW, COD determination, and HPLC analyses.

### 3.6. 2-PE Extraction and Purification from Fermentation Broth

2-PE extraction from fermentation broth was performed as described previously with minor modifications [37]. After removing of yeast biomass, the obtained fermentation broth was mixed with cold (4 °C) ethyl acetate in a ratio of 2:1 (Chempur, Piekary Śląskie, Poland). The whole was gently shaken for 3 min, since vigorous shaking caused the formation of emulsion and did not improve extraction efficiency. After the separation of phases, the organic phase was poured into the flask and subsequently dried using anhydrous magnesium sulfate. The drying agent was removed by filtration and ethyl acetate was evaporated. The purity of 2-PE was estimated by GC.

### 3.7. Analytical Methods

2-PE and L-phe concentration in fermentation broth was determined by a high-performance liquid chromatography, HPLC (SYKAM chromatograph, Sykam GmbH, Eresing, Germany), with a DAD detector and a Cosmosil 5C18-MS-II reversed-phase column (250 × 4.6 mm, 118.5 μm). An isocratic method comprising 70:30 water/acetonitrile (ACN) was used. A DAD detector was set at a wavelength of 259 nm.

Lactose and ethanol content was determined by HPLC coupled with RI detector and a SETREX IEX H+ column (300 × 8 mm column, Polymer IEX H form, 8 μm) under thermostatic control at 35 °C. RI detector was also set at 35 °C in order to avoid fluctuations in detector responses. Samples were eluted isocratically using 9 mM H_2_SO_4_ as the mobile phase, at a flow rate of 1 mL min^−1^.

To calculate the dry cell weight (DCW), 10 mL aliquots were centrifuged at 5000× *g* for 2 min; supernatants were transferred to other tubes and yeast pellets were dried at 85 °C until reaching constant weight. Supernatants were then used to determine chemical oxygen demand (COD) with a NANOCOLOR CSB 600 kit (Macherey-Nagel, Düren, Germany) according to the DIN ISO 15705–H45 method.

The cell density in liquid medium samples was monitored by measuring turbidity at 600 nm (OD_600_) using a VIS-7220G spectrophotometer (Beijing Rayleigh Analytical Instrument Corporation Co., Ltd., Beijing, China).

### 3.8. Calculations

The parameters of the 2-PE bioproduction were calculated from the following equations:

(1) $$\mathrm{Specific}\;\mathrm{growth}\;\mathrm{rate}\;(\mu )\;\;\mu \;=\frac{1}{OD_{600}}\frac{dOD_{600}}{dt}\;\left(h^{-1}\right)\;$$

(2) $$\text{Space-time-time}\;(P_{2-PE})\;\;P_{2-PE}\;=\frac{\Delta C_{2-PE}}{\Delta t}\;\left(\;{\mathrm{mg}\;L}^{-1}h^{-1}\right)$$

(3) $$\mathrm{Specific}\;\mathrm{product}\;\mathrm{yield}\;(Y_{2-PE})\;\;Y_{2-PE}\;=\frac{\Delta C_{2-PE}}{\Delta C_{2-PE}^{th}}*100\;\left(\%\right)$$

(4) $$\mathrm{Product}\;\mathrm{yield}\;\mathrm{per}\;\text{L-phe}\;(Y_{P/L-phe0})\;\;Y_{P/L-phe0}\;=\frac{\Delta C_{2-PE}}{\Delta C_{L-phe}}\;\left(-\right)$$

(5) $$\text{L-phenylalanine}\;\mathrm{usage}\;(Z_{L-phe})\;\;Z_{L-phe}\;=\frac{1-\Delta C_{L-phe}}{C_{L-phe0}}*100\;\left(\%\right)$$

where OD_600_ is a turbidity at *λ* = 600 nm, $\Delta C_{2-PE}$ is the ratio of the achieved concentration of the product (2-PE), $C_{2-PE}^{th}$ is the maximum theoretical concentration of the product, which can be achieved as a result of the biotransformation of *L-Phe*, $C_{L-phe0}$ is an initial concentration of *L-phe*, and $C_{L-phe}$ is a concentration of *L-phe* at the end of the culture.

### 3.9. Statistical Data Analyses

All analyses were performed at least in three biological repetitions and the data are presented as the mean ± standard deviation (SD). Statistical comparisons were performed between groups using Student unpaired t-tests; *p* < 0.05 was the criterion for statistical significance.

## 4. Conclusions

In this work, three newly isolated yeasts assigned to *K. marxianus* and *K. lactis* species have been evaluated for 2-PE production. Although all tested microorganisms produce a similar amount of 2-PE (approx. 2 g L^−1^) through L-phe biotransformation in whey permeate-based media, the *K. marxianus* strains seem to be better producers as they are more resistant to the increasing concentration of 2-PE. Thus, further work has been undertaken to draw up a whole procedure of 2-PE production only with *K. marxianus* WUT240, firstly under a batch mode and, subsequently, in a continuous system. This has resulted in a laboratory scale-technology development, and this is the first report documenting a complete methodology for 2-PE bioproduction on whey permeate under continuous mode, starting from biotransformation to purification of the final product. The use of yeast with GRAS and QPS status as well as whey permeate as a culture medium or ethyl acetate as an extractant creates an opportunity to provide a solution which could be environmentally friendly. In the designed technological process, four main streams are generated: yeast biomass, 2-PE, ethyl acetate, and residual aqueous phase after extraction. Both 2-PE and yeast biomass are end-products, with various applications in the food, feed, cosmetic, or pharmaceutical sectors. Ethyl acetate, after regenerative distillation, can be used repeatedly for the extraction of 2-PE, thus eliminating the need for its development. The latter aqueous phase with residual ethyl acetate content can be preheated to 77 °C to evaporate ethyl acetate and subsequently for re-use for 2-PE extraction. Impoverished water can be used for the next bioprocess.

## Figures and Tables

**Table 1:** 2-PE concentration [g L^−1^] in batch cultures of *K. lactis* WUT175 and *K. marxianus* WUT216 and WUT240 strains conducted in medium 8 at 25, 30 or 37 °C for 48 h.

Growth Temperature	Time	WUT175	WUT216	WUT240
25 °C	24 h	0.89 ± 0.04	1.13 ± 0.09	1.62 ± 0.05
	48 h	1.63 ± 0.01	2.74 ± 0.34	2.25 ± 0.04
30 °C	24 h	0.77 ± 0.01	1.58 ± 0.08	1.85 ± 0.07
	48 h	1.24 ± 0.03	2.21 ± 0.07	2.14 ± 0.01
37 °C	24 h	- ^1^	1.57 ± 0.02	1.85 ± 0.07
	48 h	- ^1^	1.85 ± 0.02	1.82 ± 0.01

^1^ not tested, because WUT175 does not grow at 37 °C.

**Table 2:** 2-PE concentrations [g L^−1^] achieved in batch cultures of *K. lactis* WUT175 and *K. marxianus* WUT216 and WUT240 strains in two whey permeate based media (WP1 and WP2) conducted for 48 h at 25 °C and 30 °C. Analyses were performed in triplicate and data are presented as the mean ± SD.

Medium	Growth Temperature	Time	WUT175	WUT216	WUT240
WP1	25 °C	24 h	1.37 ± 0.09	1.14 ± 0.28	1.94 ± 0.08
		48 h	1.78 ± 0.19	1.65 ± 0.24	1.99 ± 0.08
	30 °C	24 h	1.48 ± 0.01	1.59 ± 0.17	1.00 ± 0.04
		48 h	1.65 ± 0.01	1.90 ± 0.21	1.16 ± 0.05
WP2	25 °C	24 h	1.97 ± 0.12	1.08 ± 0.03	1.34 ± 0.40
		48 h	2.12 ± 0.19	1.00 ± 0.03	2.14 ± 0.40
	30 °C	24 h	1.74 ± 0.07	1.14 ± 0.07	1.68 ± 0.13
		48 h	1.87 ± 0.05	1.64 ± 0.19	2.07 ± 0.32

**Table 3:** Parameters of 48 h batch cultures of *K. marxianus* WUT240 producing 2-PE in WP2 medium in a 4.8 L bioreactor. μ—specific growth rate; P_2-PE_—space-time-yield, Y_2-PE_—specific product yield; Y_P/L-phe0_—product yield per L-phe, Z_L-phe_—L-phenylalanine usage, nd—not detected.

Parameter	Batch 1	Batch 2	Batch 3	Batch 4	Batch 5
final OD_600_ (−)	6.84 ± 0.02	6.96 ± 0.08	8.39 ± 0.02	7.83 ± 0.01	6.26 ± 0.07
μ (h^−1^)	0.25 ± 0.01	0.26 ± 0.02	0.17 ± 0.01	0.35 ± 0.01	0.36 ± 0.00
2-PE (g L^−1^)	1.66 ± 0.01	1.76 ± 0.03	1.80 ± 0.05	2.10 ± 0.11	2.59 ± 0.15
L-phe (g L^−1^)	2.64 ± 0.08	2.56 ± 0.04	2.47 ± 0.18	2.19 ± 0.18	1.88 ± 0.08
lactose (g L^−1^)	nd	nd	nd	nd	nd
ethanol (g L^−1^)	6.23 ± 0.09	5.07 ± 0.01	nd	nd	3.65 ± 0.26
P_2-PE_ (mg L^−1^ h^−1^)	34.58	36.67	37.50	43.75	53.96
Y_2-PE_ (−)	0.45	0.48	0.49	0.47	0.70
Y_P/L-phe0_ (−)	0.33	0.35	0.36	0.42	0.52
Y_P/L-phe0_ (−)	0.33	0.35	0.36	0.42	0.52
Z_L-phe_ (%)	47.20	48.80	50.60	58.00	62.40

**Table 4:** Parameters of 72 h continuous cultures of *K. marxianus* WUT240 producing 2-PE in WP2 medium at 30 °C.

Parameter	Continuous 1 (0.05 h^−1^ Dilution Rate)	Continuous 2 (0.1 h^−1^ Dilution Rate)
final OD_600_ (−)	2.65 ± 0.06	2.86 ± 0.14
DCW (g L^−1^)	1.30 ± 0.00	1.30 ± 0.04
μ (h^−1^)	0.28 ± 0.02	0.36 ± 0.02
2-PE (g L^−1^)	1.15 ± 0.12	0.85 ± 0.07
amount of 2-PE (g) ^1^	8.29 ± 0.82	5.63 ± 0.54
L-phe (g L^−1^)	0.47 ± 0.03	0.08 ± 0.04
lactose (g L^−1^)	nd	nd
ethanol (g L^−1^)	nd	nd
P_2-PE_ (mg L^−1^ h^−1^)	57.50	39.10
Y_2-PE_ (−)	1.12	0.76
Y_P/L-phe0_ (−)	0.33	0.35
Y_P/L-phe0_ (−)	0.83	0.56
Z_L-phe_ (%)	90.6	85.0

^1^ recovered from fermentation broth. μ—specific growth rate; P_2-PE_—space-time-yield, Y_2-PE_—specific product yield; Y_P/L-phe0_—product yield per L-phe, Z_L-phe_—L-phenylalanine usage, nd—not detected.

**Table 5:** Media composition.

Medium	Composition	Reference
SAB	5 g L^−1^ meat peptone, 5 g L^−1^ casein peptone, 20 g L^−1^ glucose (Merck, Darmstadt) pH 5.6	-
8	15 g L^−1^ glucose, 8 g L^−1^ sucrose, 5 g L^−1^ L-Phe (BioShop, Burlington, Canada; purity min. 98.5%), 0.5 g L^−1^ MgSO4, 1.7 g L^−1^ YNB without amino acids and ammonium sulfate (Conda, Madrid, Spain), pH 4.6	[36]
WP1	15 g L^−1^ whey permeate ^1^ (SM Mlekovita, Wysokie Mazowieckie, Poland), 5 g L^−1^ L-phe	this work
WP2	25 g L^−1^ whey permeate^1^, 5 g L^−1^ L-phe	this work

^1^ spray dried whey permeate achieved after ultrafiltration of whey, composed of ~80% lactose, 3–5% protein, moisture max. 4%, max. 1.5% fat, and max. 8.5% ashes.

**Table 6:** Parameters of five batch cultures in a 4.8 L bioreactor.

Parameter	Batch 1	Batch 2	Batch 3	Batch 4	Batch 5
inoculum titer	2%	2%	2%	2%	2%
bioreactor working volume	2 L	2 L	2 L	2 L	2 L
temperature	25 °C	25 °C	25 °C	30 °C	30 °C
initial pH	5	5	5	5	5
air flow rate	1.5 L min^−1^	3.0 L min^−1^	3.0 L min^−1^	3.0 L min^−1^	1.5 L min^−1^
stirring speed	250 rpm	250 rpm	500 rpm	500 rpm	250 rpm

## Data Availability

Yeast strains are deposited in a publicly available Warsaw University of Technology Yeast Collection (WUT YC), https://wutyeastcollection.pw.edu.pl/, accessed on 3 November 2021. DNA sequences of the ITS1-5.8S-ITS2 regions and the D1/D2 domains of 26S rDNA are deposited in the NCBI GenBank database under numbers: OK093387, OK093398, OK093388, OK093399, OK093389, OK093400.

## Supplementary Materials

The following are available online, Table S1: Source of *Kluyveromyces lactis* WUT175, *Kluyveromyces marxianus* WUT216, and *K. marxianus* WUT240 strains, and their physiological characteristics.

## Sample Availability

Samples of the yeast strains are available from the authors.
