# Supplementary material for: Development of a Continuous System for 2-Phenylethanol Bioproduction by Yeast on Whey Permeate-Based Medium
Source: Molecules. 2021 Dec 6;26(23):7388. doi: 10.3390/molecules26237388 (PMC8658864; doi:10.3390/molecules26237388)
Supplement: Supplementary file 1 [file molecules-26-07388-s001.zip › molecules-1473568-supplementary.pdf]

Supplementary Material

Development of a Continuous System for 2-phenylethanol Bioproduction by Yeast on Whey Permeate-Based Medium

Karolina Dreżek [1], Joanna Kozłowska [1], Anna Detman [2] and Jolanta Mierzejewska [1,*]

[1] Chair of Drug and Cosmetics Biotechnology, Faculty of Chemistry, Warsaw University of Technology, 00-664 Warsaw, Poland; karolina.drezek@pw.edu.pl (K.D.); kozlowska.joanna.1997@gmail.com (J.K.)
[2] Laboratory of White Biotechnology, Institute of Biochemistry and Biophysics, Polish Academy of Sciences, 02-106 Warsaw, Poland; annadetman@ibb.waw.pl
* Correspondence: jolanta.mierzejewska@pw.edu.pl

Table S1. Source of *Kluyveromyces lactis* WUT175, *Kluyveromyces marxianus* WUT216 and *K. marxianus* WUT240 strains, and their physiological characteristics.

**Strain Number**	**WUT175**	**WUT216**	**WUT240**
Source	mare's milk, Kyrgyzstan	kefyr, Poland	mare’s milk, Kyrgyzstan
GeneBank accesion numbers			
ITS1- 5.8S-ITS2 rRNA	OK093387	OK093388	OK093389
26S rRNA	OK093398	OK093399	OK093400
		**Physiological Characteristics**	
Assimilation-growth			
D-glucose	+	+	+
D-xylose	-	+	+
D-galactose	+	+	+
Lactose	+	+	+
Maltose	+	+	d
Sucrose	+	+	+
Glycerol	+	+	-
Ethanol	+	+	+
Temperature-growth			
	10–30 °C	10–42 °C	15–42 °C
Enzymatic Activity			
Lipolytic	+	+	-
Amylolytic	-	-	-
Proteolytic	-	-	-
Cellulolytic	-	-	-

„+ / -” – growth/ no growth, „d”- delayed growth.
